# Host factors that shape the bacterial community structure on scalp hair shaft

**DOI:** 10.1038/s41598-021-96767-w

**Published:** 2021-09-06

**Authors:** Kota Watanabe, Azusa Yamada, Yuri Nishi, Yukihiro Tashiro, Kenji Sakai

**Affiliations:** 1 Laboratory of Soil and Environmental Microbiology, Division of Systems Bioengineering, Department of Bioscience and Biotechnology, Faculty of Agriculture, Graduate School, Kyushu University, Motooka 744, Nishi-ku, Fukuoka, 819-0395 Japan; 2 Laboratory of Microbial Environmental Protection, Tropical Microbiology Unit, Center for International Education and Research of Agriculture, Faculty of Agriculture, Kyushu University, Motooka 744, Nishi-ku, Fukuoka, 819-0395 Japan

**Keywords:** Microbiology, Bacteria

## Abstract

In this study, we performed 16S rRNA amplicon sequencing analysis of scalp hair shaft from 109 volunteers, who were surveyed using a questionnaire about daily scalp hair care, and employed multiple statistical analyses to elucidate the factors that contribute to the formation of bacterial community structures on scalp hair shaft. Scalp hair microbiota were found to be specific for each individual. Their microbiota were clearly divided into two clusters. Genus level richness of *Pseudomonas* (Ps) and *Cutibacterium* (Cu) contributed to the clusters. The clusters around *Pseudomonas* and *Cutibacterium* were named Ps-type and Cu-type, respectively. The host gender influenced the bacterial cell numbers of the major genera that included *Cutibacterium*, *Lawsonella*, *Moraxella*, and *Staphylococcus* on scalp hair shaft. In addition to host intrinsic factors, extrinsic factors such as hair styling and colouring affected the bacterial cell numbers of the major genera. These factors and chemical treatments, such as bleaching and perming, also affected the Ps-type to Cu-type ratios. These results suggest that bacterial community structures on scalp hair shaft are influenced by both intrinsic and extrinsic factors.

## Introduction

Studies of the human microbiome, using 16S amplicon sequencing, have revealed the site-specific bacterial community structures of not only internal organs, such as the gut and mouth, but also external organs of the body, such as skin^1,2^. The human skin is the largest organ of the body, and its surface is colonised by a wide variety of microbes^3–7^. Skin microbiota at each body site are characterised by distinct community structures^3,8–10^. Variations in such bacterial community structures are associated with the ecological zones of the skin, including the sebaceous, dry, or moist environments^4,5^. Differences in the bacterial communities on skin are also related to host intrinsic factors such as gender, age, and ethnicity, as well as extrinsic factors such as lifestyle and living environment^11–13^.

The human skin microbiome has been analysed on a large scale, but human scalp hair, present on the skin, has not. There are several skin sites with hair, including the scalp, pubis, armpits, and legs. Tridico et al. analysed bacterial community structures on pubic and scalp hair and showed that they were only different in females^14^. We have previously reported the special features of bacterial community structures on scalp hair^15–17^. A specific individual bacterial community structure on scalp hair could be identified using the terminal restriction fragment length polymorphism method^15^. Furthermore, we analysed the bacterial community structure on portions of human scalp hair shafts and roots from six individuals, using 16S amplicon sequencing, and found that the major bacteria on human scalp hair shafts are indigenous and derived from hair roots^16^. In a comparative analysis of bacterial community structures on scalp hair shaft and scalp skin, scalp hair shaft contained the hair-specific genus, *Pseudomonas*, and the skin-derived genera *Cutibacterium* and *Staphylococcus*, which were distinguishable from other human skin microbiomes^17^. These studies piqued our interest in why the bacterial community structure on scalp hair shaft is distinguishable, and different from that on the scalp skin. Similar to the skin microbiome^13^, the structure of human scalp hair microbiota may be influenced by multiple intrinsic factors, such as gender, age, and skin physiological parameters, and extrinsic factors, such as hair washing, styling, and chemical treatments.

In this study, we combined 16S amplicon analysis with a questionnaire about daily hair care and performed multiple statistical analyses to elucidate the factors that contribute to the formation of bacterial community structures on scalp hair shaft.

## Results

### UniFrac analysis on scalp hair shaft of individuals

**Figure 1:**
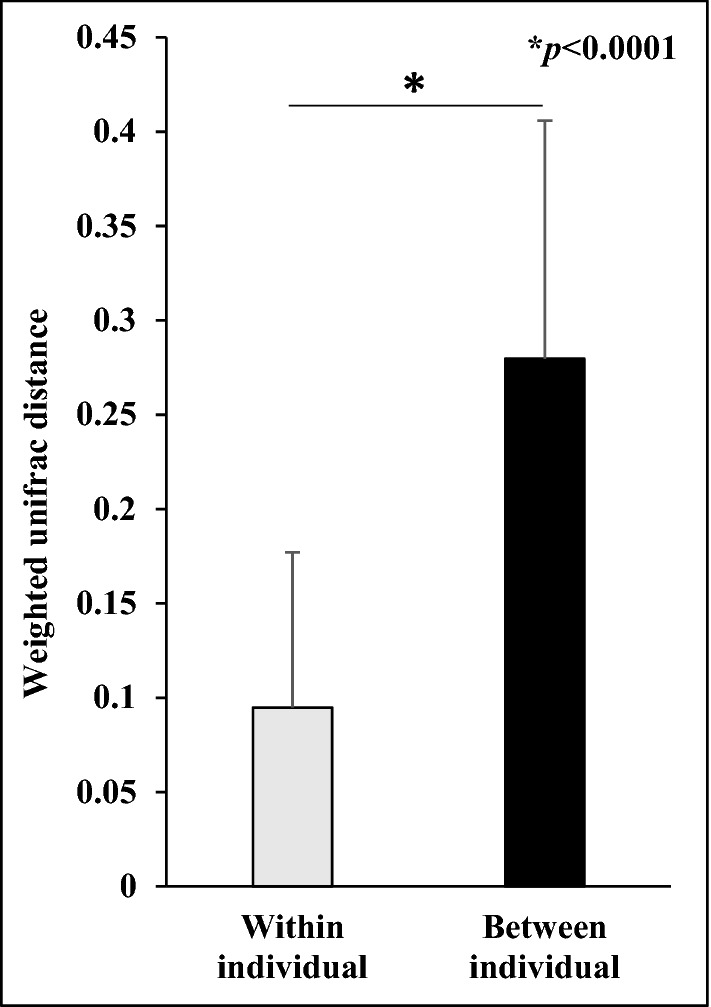
Comparison of weighted Unifrac distances within and between individual samples.

UniFrac analysis was performed to compare the overall structural similarity and variation in the bacterial community between individual samples using the weighted UniFrac distance (Fig. 1). The weighted UniFrac distances of samples within individuals were significantly lower (*p* < 0.0001) than those between individuals. This indicated that the bacterial community structure on scalp hair shaft was more similar within individuals than between individuals and specific in each individual. Therefore, the results of three samples from each individual were averaged and used for clustering analysis.

### Bacterial community structures on scalp hair shaft at phylum and genus level

**Figure 2:**
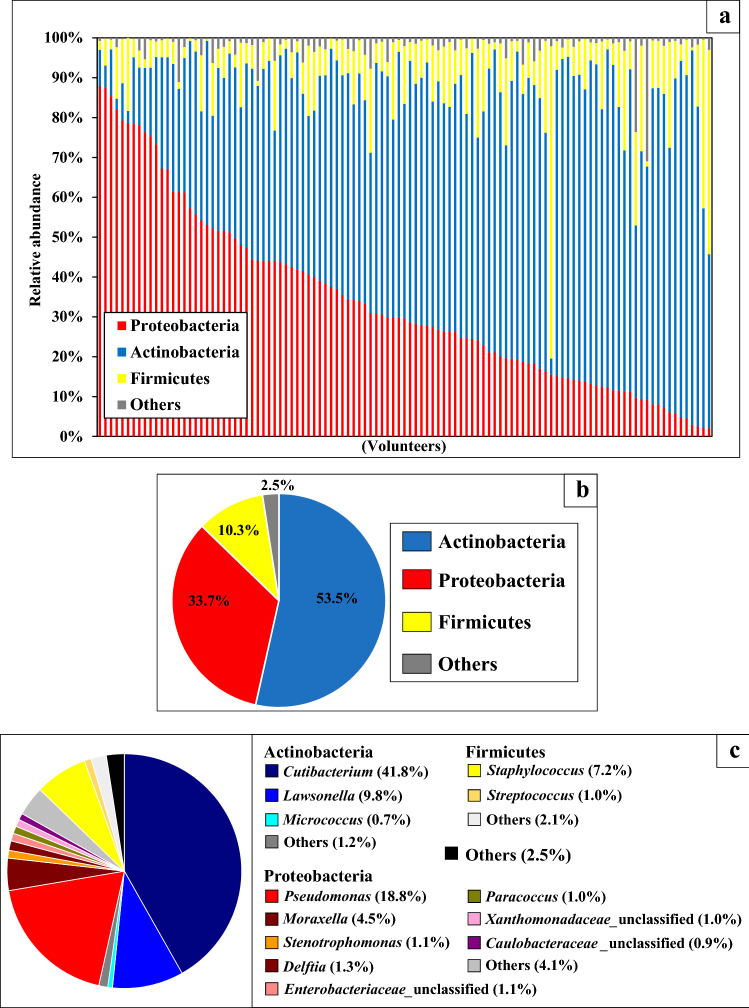
(**a**) The relative abundance of major phyla in the bacterial community structure on each volunteer of scalp hair shaft. Volunteers were arranged according to relative abundance of Proteobacteria. (**b**) The average relative abundances of the major phyla in bacterial community structure on scalp hair shaft. (**c**) The average relative abundances of the major genera in bacterial community structure on scalp hair shaft.

Three major phyla, Actinobacteria, Proteobacteria, and Firmicutes, were found in all the volunteers, with average abundances of 53.5%, 33.7%, and 10.3%, respectively (Fig. 2a,b). The relative abundances of the major phyla varied among individuals. At the genus level, the Actinobacteria *Cutibacterium* and *Lawsonella*, Proteobacteria *Pseudomonas* and *Moraxella*, and Firmicute *Staphylococcus* were found in all the volunteers, with an average abundance of 41.8%, 9.8%, 18.8%, 4.5%, and 7.2%, respectively (Fig. 2c).

Proteobacteria and Actinobacteria tended to compete for dominance (Fig. 2a). Multiple regression analysis was performed to determine the correlation between the major bacterial phyla (Table 1). A strong negative correlation was observed between the relative abundances of Proteobacteria and Actinobacteria (R = − 0.87). Similarly, a multiple regression analysis at the genus level showed a strong negative correlation between the relative abundances of *Pseudomonas* among Proteobacteria and *Cutibacterium* among Actinobacteria (R = − 0.66). Similar to the competition of Proteobacteria and Actinobacteria at the phylum level (Fig. 2a), this correlation suggested that *Pseudomonas* and *Cutibacterium* competed for dominance.

**Table 1:**
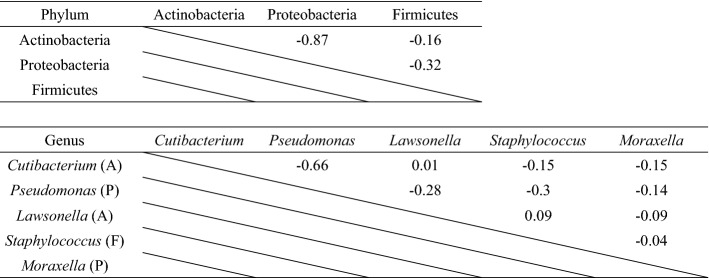
Correlation analysis of relative abundance between major bacterial phyla and genera. These figures show correlation coefficient. (A): phylum Actinobacteria, (P): phylum Proteobacteria, (F): phylum Firmicutes.

### Clustering analysis of bacterial community structures on scalp hair shaft at phylum and genus level

**Figure 3:**
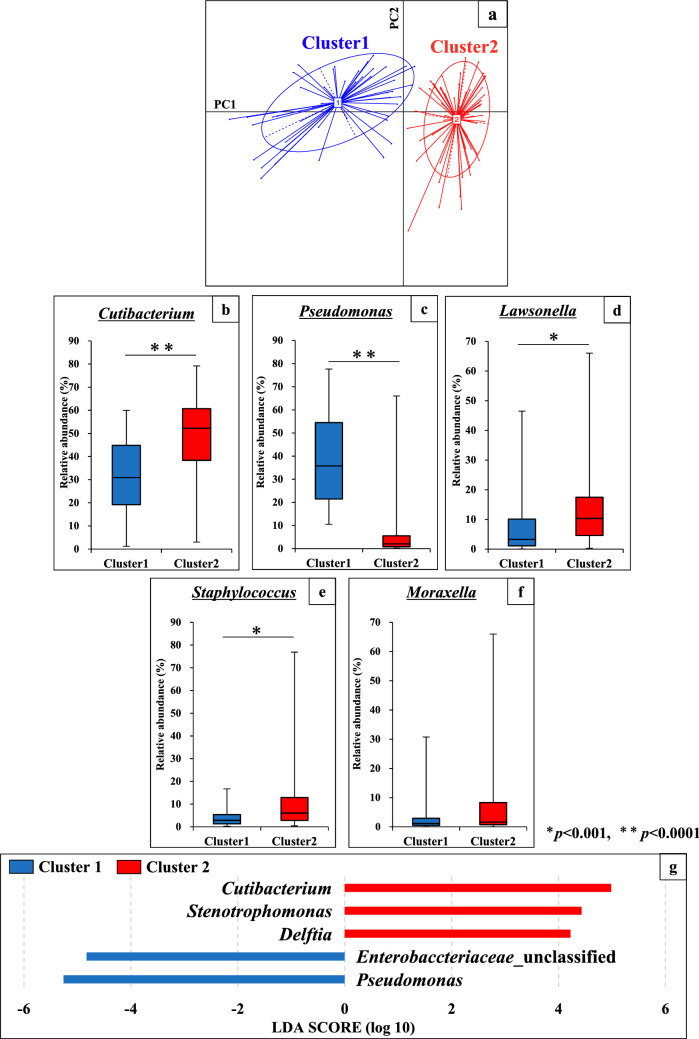
Analysis of bacterial community structure in scalp hair shaft samples at genus level. (**a**) Clustering of the 109 volunteers in principal component analysis at the genus level. The 109 samples were clustered using the partition around medoids (PAM) clustering. The two clusters were divided into a PC1-negative region (cluster 1) and a PC1-positive region (cluster 2). (**b**–**f**) Comparison of relative abundance of major five genera between cluster 1 and cluster 2 in scalp hair shaft samples. (**g**) Histogram of the Linear discriminant analysis (LDA) scores computed for microbial genera differentially abundant between cluster 1 and cluster 2 in scalp hair shaft samples.

Clustering of 109 individual samples was attempted at each taxonomic level to classify the bacterial community structure on scalp hair shaft. Herein, we present the results at the genus level (Fig. 3). The plots were clearly divided into two clusters (Clusters 1 and 2) in principal component analysis at the genus level (Fig. 3a). To investigate the differences in bacterial community structure between the two clusters, we compared the relative abundances of the major bacteria at the genus level. Among the five major genera, the relative abundances of *Cutibacterium*, *Pseudomonas*, *Lawsonella*, and *Staphylococcus*, unlike that of *Moraxella*, were significantly different in the two clusters (Fig. 3b–f). Furthermore, we investigated the bacterial factors that contribute to cluster formation using the linear discriminant analysis effect size (LEfSe) tool. At the genus level, *Pseudomonas* and *Cutibacterium* had the highest Linear discriminant analysis (LDA) scores in Clusters 1 and 2, respectively (Fig. 3g). Although *Stenotrophomonas*, *Delftia*, and *Enterobacteriaceae* were also found, the relative abundance of each bacterial genus and family was low (1.1%, 1.3%, and 0.2%, respectively) (Fig. 2c). Hereinafter, the *Pseudomonas-* and *Cutibacterium*-defined clusters are referred to as ‘*Pseudomonas* (Ps)-type’ and ‘*Cutibacterium* (Cu)-type’, respectively.

### Quantification of bacterial cell number on scalp hair shaft

Bacterial cell numbers in 327 scalp hair shaft samples derived from 109 volunteers were quantified (Table 2). The average hair shaft length and diameter were 15.9 (± 9.9) cm and 89.0 (± 14.7) μm, respectively. The bacterial cell number per cm^2^ of hair shaft was 3.5 (± 7.6) × 10^5^, with maximum and minimum numbers of 7.4 × 10^6^ and 4.2 × 10^3^, respectively. While there was a significant difference in hair shaft length between genders (ANOVA, *p* < 0.05), there was no significant difference in bacterial cell number and hair shaft diameter. In addition, no correlation between hair shaft length or diameter and bacterial cell number per cm^2^ was observed (R = − 0.04 and − 0.09, respectively) (Supplementary Fig. S1a,b).

**Table 2:**
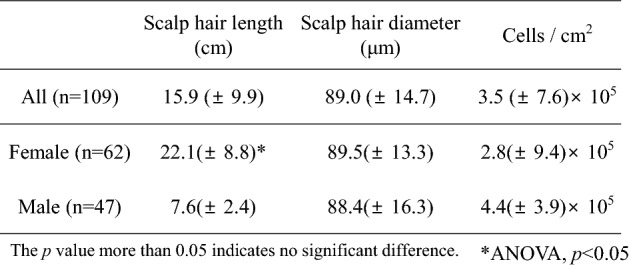
Measurement of the length and diameter of scalp hair shaft, and quantification of the bacterial cell number on scalp hair shafts by qPCR of the 16S rRNA gene copy. * indicates significantly high number between female and male.

### Association between host intrinsic and extrinsic factors and bacterial community structure on scalp hair shaft

To investigate the factors that influence the formation of bacterial community structures, we analysed host intrinsic (gender) and extrinsic factors (Tables 3 and 4, and Supplementary Table S2). Although the age and ethnicity of the volunteers were also host intrinsic factors, there were no significant differences in bacterial community structure with respect to these (Supplementary Table S2). In addition, since these intrinsic factors had a bias in the number of volunteers in this study, we analysed gender as a key host intrinsic factor even though the bacterial cell numbers were not significantly different between genders, as described above (Table 2). Table 3 shows the two indices of alpha diversity and cell number of major bacteria at the genus level for each gender. There were no significant differences in alpha diversity indices between the genders. However, bacterial cell numbers of *Cutibacterium*, *Lawsonella*, *Moraxella*, and *Staphylococcus* were significantly higher in males than in females. On the other hand, the bacterial cell number of *Pseudomonas* was higher in females than in males.

**Table 3:**
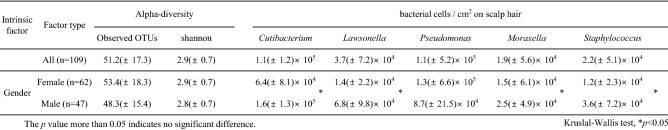
Alpha diversity and absolute number of the major bacterial genera between genders. *p* value more than 0.05 was showed no significant different. * indicates significantly high numbers between female and male.

**Table 4:**
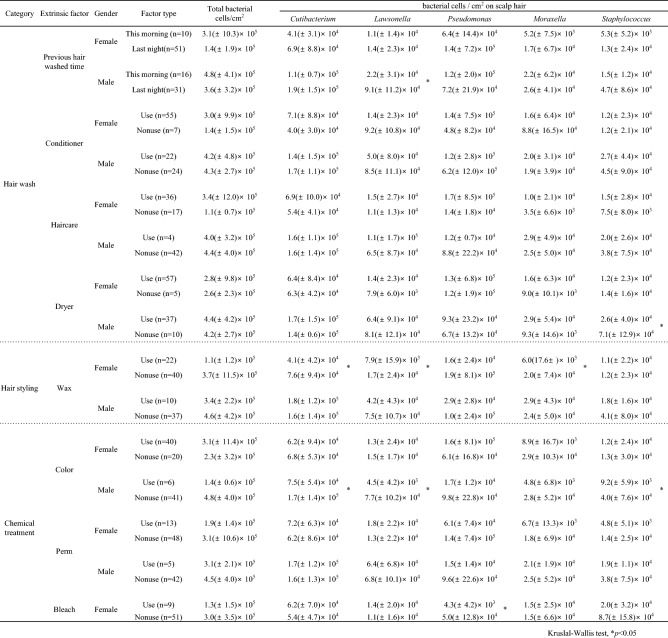
Absolute number of major the bacterial genera at each comparative category and each gender. ** and * indicate significantly high and low numbers among extrinsic factors, respectively.

Supplementary Table S3 shows the cell number of major bacteria at the genus level for each host extrinsic factor. There were significant differences with respect to some extrinsic factors, including treatment with conditioner, hair care, and hair colour. Three major bacterial genera (*Cutibacterium*, *Lawsonella*, and *Staphylococcus*) were significantly different in all categories. The genera *Pseudomonas* and *Moraxella* were not significantly different in any category. Similarity percentage (SIMPER) analysis (Supplementary Table S4) revealed that the five major bacterial genera of *Pseudomonas*, *Cutibacterium*, *Lawsonella*, *Staphylococcus*, and *Moraxella* differed meaningfully in the community structure for each extrinsic factor with contributions of 11.0–12.5%, 10.4–12.2%, 4.9–6.1%, 3.3–4.5%, and 2.6–3.4%, respectively. However, since the influence of gender was considered, extrinsic factors were analysed for each gender (Table 4). In females, treatment with hair wax resulted in significantly lower bacterial cell numbers of *Cutibacterium*, *Lawsonella*, and *Moraxella* than those without treatment. In addition, treatment with hair bleach in females resulted in a significantly lower *Pseudomonas* cell number than those without treatment. In males, treatment with hair colour resulted in significantly lower cell numbers of *Cutibacterium*, *Lawsonella*, and *Staphylococcus* than those without treatment. Treatment with a hair dryer in males resulted in a significantly lower *Staphylococcus* cell number than those without treatment. Individuals who had washed their hair that morning in males had significantly lower *Lawsonella* cell numbers than those who had washed their hair the previous night.

**Figure 4:**
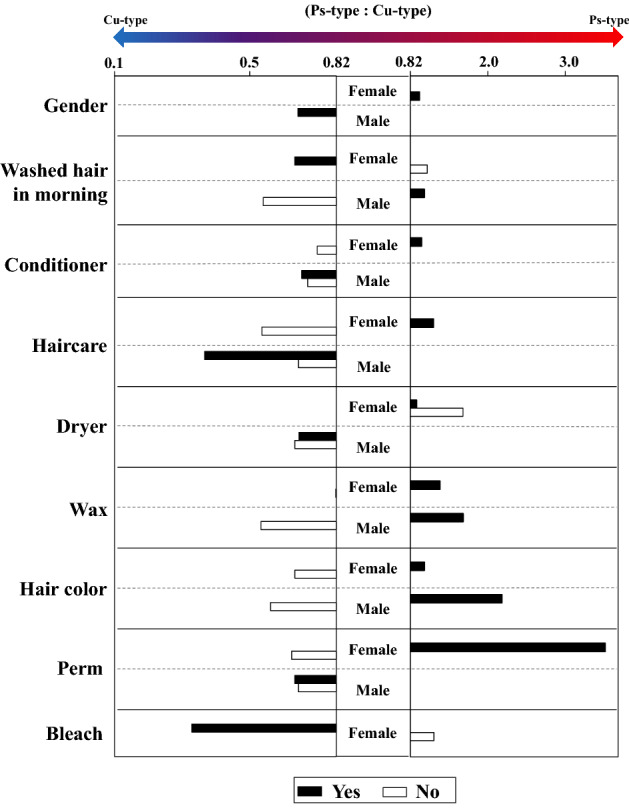
Differences in the ratio of Ps-type and Cu-type between extrinsic factors analysed for each gender (*p ≤ 0.05). The reference value of the ratio of Cu-type to Ps-type for all the subjects was 0.82. Values > 0.82 indicated Ps-type, and those < 0.82 indicated a Cu-type.

Furthermore, the ratio of Ps-type to Cu-type for each host factor was analysed (Fig. 4). The reference value of the ratio of Cu-type to Ps-type for all the subjects was 0.82. Females exhibited a slightly higher Ps-type to Cu-type ratio, whereas males exhibited a higher Cu-type to Ps-type ratio. Some extrinsic factors such as treatment with hair conditioner in females, haircare agents in females, hair wax, hair colour, and hair perm in females exhibited higher Ps-type to Cu-type ratios. In addition, treatment with hair bleach in females resulted in a higher Cu-type to Ps-type ratio. In particular, treatment with hair wax in males, hair colour in males, perm in females, and hair bleach in females caused large differences in the Cu-type to Ps-type ratio (greater than 0.8). On the other hand, treatment with hair conditioners, hair care agents, and perms had different effects on the Ps-type to Cu-type ratio depending on the gender. The results suggested that host intrinsic and extrinsic factors affected the bacterial community structure on scalp hair shaft, since bacterial cell numbers of the major genera and the ratio of cluster types were influenced by these factors.

## Discussion

Recent studies of the human microbiome on scalp hair have shown that bacterial community structure varies between individuals and is distinguishable from that of other human skin microbiomes^14–17^. This study provided the first insights into the factors that contribute to the formation of bacterial community structures on scalp hair shaft. We found four characteristics of bacterial community structures on scalp hair shaft. Firstly, they were specific for each individual. Secondly, they were clustered into two groups representing *Pseudomonas* (Cluster: Ps-type) and *Cutibacterium* (Cluster: Cu-type), and these two genera competed for higher relative abundance. Thirdly, the cell numbers of the major bacterial genera were significantly different between genders (intrinsic factor). Fourthly, extrinsic factors such as hair treatment with harsh chemicals affected the cell numbers of major bacterial genera and the Ps-type to Cu-type ratio. We discuss each result individually.

Firstly, we compared the similarity of the bacterial community structure on scalp hair shaft within and between individuals, and found that weighted UniFrac distances within individuals were significantly lower than those between individuals (Fig. 1). Costello et al. surveyed bacteria from up to 27 sites, including scalp hair, and reported that interpersonal variability was high, whereas individuals exhibited minimal temporal variability^3^. In addition, Williams et al. analysed the bacterial community structure on pubic hair, and reported that the weighted UniFrac distance between individuals was greater than that within individuals^18,19^. This indicated that the bacterial community structure on scalp hair shaft was stable and individually established. This also suggested that intrinsic factors such as age and gender, and extrinsic factors such as lifestyle and haircare might affect the bacterial community structure of scalp hair shaft.

Secondly, we identified five genera, *Cutibacterium* (avg. 42%), *Pseudomonas* (avg. 19%), *Lawsonella* (avg. 10%), *Staphylococcus* (avg. 7%), and *Moraxella* (avg. 4%), that were present in all individuals, with an average abundance of more than 4% (Fig. 2c). A strong negative correlation was observed between the relative abundances of *Pseudomonas* and *Cutibacterium* (R = − 0.66) (Table 1). Furthermore, the partition around medoids (PAM) clustering and LEfSe methods indicated that bacterial community structures on scalp hair shafts at the genus level were divided into *Pseudomonas* (Ps)-type and *Cutibacterium* (Cu)-type (Fig. 2a). It has been reported that gut microbiota can be divided into two or three clusters^20,21^. Variations in the cutaneous microbiota of healthy volunteers were associated with the ecological zones of the skin, including the sebaceous, dry, and moist environments^10^. It is unclear why bacterial community structures on scalp hair shaft were divided into two clusters, although our results showed the influence of gender (intrinsic factor) and hair treatment (extrinsic factor). Each factor is explained in detail later.

Thirdly, we confirmed that the average bacterial cell number per cm^2^ of scalp hair shaft was stable at approximately 3.5 (± 7.6) × 10^5^ by estimating the 16S rRNA gene copy number (Table 2), which was consistent with that obtained in previous studies^16,17,22^. These results suggest that scalp hair shafts have a load of 10^5^ bacterial cells without exception, and that shampooing or chemical treatment of scalp hair shaft would not significantly affect the total cell number (Table 4). The bacterial cell numbers of four major genera, except that of *Pseudomonas*, were significantly different between genders (Table 3). Cutaneous environments such as sweat, sebum, and hormone production sites were different between genders^9,23^, which would affect the diversity of bacterial community structure on human skin between genders^9^. To the best of knowledge, a relationship between environments of scalp skin and bacterial community structure between genders has not been reported. Because many bacterial species were reported to be shared between scalp skin and scalp hair shaft^17^, these environmental differences of scalp skin may be related to microbial differences in scalp hair shaft. Further research on intrinsic factors showed no difference in the cell numbers of major bacterial genera with respect to age and ethnicity (Supplementary Table S2). In contrast, Perez-Perez et al. analysed skin microbiota present in three skin sites (forearm, axilla, and scalp) from six ethnic groups living in New York City and reported that ethnicity is a secondary factor in determining cutaneous microbiota composition^12^. In addition, Shibagaki et al. reported that the skin microbiota present in four skin sites (scalp, forehead, cheek, and volar forearm) were different in young- and old-age groups^24^. Since the volunteers in this study were mostly Asian and in their twenties, further detailed studies are required to clarify the factors of age and ethnicity.

In addition to the host intrinsic factors, such as gender, we found an association between host extrinsic factors and the bacterial community on scalp hair shaft. We also analysed the effects of host extrinsic factors on the cell number of major bacterial genera, with special focus on the ratio of Ps-type to Cu-type clustering (Table 4, Fig. 4). Treatments with hair wax in females, bleach in females, hair colour in males, and dryers in males decreased the bacterial cell numbers of some major genera, although there were no significant differences in the total bacterial cell number (Table 4). In addition, treatments with hair perm in females, bleach in females, hair wax in males, and hair colour in males caused large differences in the Ps-type to Cu-type ratio (Fig. 4). Studies have shown that hair-wash detergents such as shampoos do not affect bacterial adherence to scalp hair shaft^16,25^. Dimitriu et al. reported that intrinsic factors such as age and ethnicity as well as extrinsic factors such as skin protection habits were predictors of microbiota composition at skin sites^13^. Hair wax is mainly composed of wax and solid lipids, and is used as a daily hair treatment for hair styling^26^. Coating the hair surface with wax may decrease the cell numbers of specific genera, including *Lawsonella*, and *Moraxella*. Furthermore, it has been reported that treatment with chemicals such as dyes and bleach damages hair fibres^27^. In particular, the cell membrane complex of scalp hair is reported to be very vulnerable to chemical treatments^28^. Our results indicated that treatment with chemicals, such as hair colour and bleach, affected the bacterial community structure of scalp hair shaft. Damage to the surface and membrane of scalp hair may affect the adhesion of major bacteria.

In conclusion, the results of this study suggested that both intrinsic factors such as gender and extrinsic factors such as daily hair styling and chemical treatments might determine the microbiota composition of scalp hair shaft. The results of our study are important for future research to improve scalp hair care and health. The scalp microbiota of volunteers with dandruff, a type of scalp disease, were reported to be in disequilibrium with respect to the proportion of the major bacteria, *Cutibacterium* and *Staphylococcus*^29–33^. Scalp hair microbiota may have to be analysed in the future. Further studies investigating the interaction between scalp hair and scalp hair-specific bacteria are currently ongoing.

## Materials and methods

### Samples and collection

Scalp hair shaft samples were collected from 109 healthy individuals of both sexes (62 females and 47 males), ranging in age from 19 to 64 years, who consented to participate in this study (Supplementary Table 1). Three scalp hair shafts were collected per person, and three samples were prepared to examine the variation within individuals. None of the volunteers was taking any medication during the experimental period. Scalp hair shaft samples were cut using sterilised scissors with nitrile gloves. After sampling, scalp hair shafts were chopped into pieces ca. 5 mm in length with scissors and placed into plastic microtubes. Subjects gave written informed consent with the approval of the Ethics Committee of the Graduate School of Bioscience and Biotechnology at Kyushu University. The methods were carried out in accordance with the approved guidelines.

When collecting scalp hair shaft, a questionnaire about scalp hair care was circulated. The results of the questionnaire for each volunteer are shown in Supplementary Table 1. We collected information on the gender and age of the volunteers (intrinsic factors) and the daily use of conditioners, haircare agents, waxes, and dryers, as well as the time of previous hair wash (extrinsic factors). In addition, we collected information on hair colour, perm, and bleach treatments up to a month before sampling.

### Extraction of bacterial DNA from scalp hair shaft

Bacterial DNA was extracted using the NucleoSpin^®^ Tissue kit (MACHEREY-NAGEL, Düren, Germany) according to the manufacturer’s instructions, with a slight modification. First, scalp hair shaft samples were immersed in 100 μl lysozyme solution (20 mg/ml lysozyme derived from egg white (Wako Pure Chemical Industries, Osaka, Japan) in 20 mM Tris–HCl and 0.2 mM EDTA, pH 8.0) for 30 min at 37 °C, as previously reported^15^, and the DNA extracts obtained (100 μl) were stored at − 20 °C until use.

### Estimation of bacterial cell number on scalp hair shaft using quantitative PCR (qPCR)

The bacterial cell numbers on the scalp hair shaft of 109 volunteers were quantified by estimating the 16S rRNA gene copy number using real-time PCR (CFX Connect™ System, BIO-RAD Laboratories, Inc., CA, USA) with universal primers for a portion of the bacterial 16S rRNA gene. We previously showed that the 16S rRNA gene copy number of scalp hair estimated by qPCR corresponds well with bacterial cell number of scalp hair obtained using direct SEM observation^16^.

Each 10 μl reaction mixture consisted of 2 μl of KOD SYBR^®^ qPCR Mix (TOYOBO Co., Ltd., Osaka, Japan), 0.1 μl of each primer [357F (5ʹ-CCT ACG GGA GGC AGC AG-3ʹ)^34^ and 518R (5ʹ-ATT ACC GCG GCT GCT GG-3ʹ)^35^], and 2 μl of bacterial DNA. The amplification programme included an initial denaturation step at 95 °C for 5 min, followed by 40 cycles of denaturation at 95 °C for 5 s, annealing at 64 °C for 20 s, and elongation at 72 °C for 20 s. DNA extract from *Escherichia coli* DH5α was used as a standard to generate a calibration curve. After amplification, the copy numbers of the 16S rRNA genes per scalp hair shaft sample were calculated per cm of scalp hair shaft and converted to per cm^2^ of scalp hair shaft. For the calculation, the following equation was used:

$${\text{Cells}}/{\text{cm}}^{{2}} \, = \,{\text{qPCR }}\,{\text{copies}}/{\text{scalp}}\,{\text{ hair }}\,{\text{shaft }}\,{\text{length }}\left( {{\text{cm}}} \right)\, \times \,{\text{scalp }}\,{\text{hair}}\,{\text{ shaft}}\,{\text{ diameter }}\left( {{\text{cm}} } \right)\, \times \,\pi .$$

The diameter of the scalp hair shaft was measured using a stereomicroscope (Stemi 305, ZEISS, Oberkochen, Germany).

### Analysis of bacterial community structures on scalp hair shaft using 16S rRNA gene sequencing

To analyse the bacterial community structures of scalp hair shaft from 109 volunteers using the MiSeq™ platform (Illumina Inc., CA, USA), a three-step PCR method was performed using the extracted DNA samples. In the first-step PCR amplification, a universal primer set for the V4 region of the bacterial 16S rRNA gene (515F, 5ʹ-GTG CCA GCM GCC GCG GTA A-3ʹ, and 806R, 5ʹ-GGA CTA CHV GGG TWT CTA AT-3ʹ)^36^ was used. As previous studies on the human microbiome on scalp hair used the primer set targeting the V4 region of the 16S rRNA gene, we selected the same hypervariable regions of the 16S rRNA gene^14,22^. The 25 μl reaction mixture consisted of 12.5 μl of Kapa HiFi HotStart Ready Mix (Kapa Biosystems Inc., Wilmington, MA, USA), 0.5 μl of each primer (10 pM), and 11.5 μl of extracted bacterial DNA. The amplification programme included an initial denaturation step at 95 °C for 3 min, followed by 40 cycles of denaturation at 98 °C for 30 s, annealing at 56 °C for 30 s, and elongation at 72 °C for 30 s. After electrophoresis through a 1.5% (w/v) agarose gel, the targeted bands were excised from the gel with sterilised cutters, and the DNA was extracted using the FastGene^®^ Gel/PCR Extraction Kit (NIPPON Genetics Co., Ltd., Tokyo, Japan), according to the manufacturer’s instructions. The DNA concentration was measured using a NanoDrop™ 2000 spectrophotometer (Thermo Fisher Scientific Inc., Waltham, MA, USA). In preparation for 16S rRNA amplicon sequencing with MiSeq, templates are given tail, adapter, and index sequences in a two-step PCR. Therefore, long-tailed primers are required for the preparation, which makes amplification difficult. We were unable to perform direct amplification using two-step PCR, probably because the amount of bacterial DNA obtained from the 3 cm scalp hair shafts was very small. Therefore, we first performed PCR using a universal primer set, without any additional sequences. Thus, we succeeded in obtaining sufficient template fragments with a minimum number of reaction cycles.

For the second-step PCR, a universal primer set for the V4 region of the bacterial 16S rRNA gene and tailed sequences for MiSeq sequencing were used (1-515F, 5ʹ-TCG TCG GCA GCG TCA GAT GTG TAT AAG AGA CAG GTG CCA GCM GCC GCG GTA A-3ʹ, and 1-806R, 5ʹ-GTC TCG TGG GCT CGG AGA TGT GTA TAA GAG ACA GGG ACT ACH VGG GTW TCT AAT-3ʹ)^37^. Although it has been reported that this primer set would poorly amplify *Propionibacterium* in human skin^38^, the results in this study showed good amplification of the predominant species on hair, *Cutibacterium acnes* (previously named *Propionibacterium acnes*). The 25 μl reaction mixture consisted of 1.0 µl of each primer (5 µM), which was heat-shocked at 95 °C for 5 min, 12.5 µl of Kapa HiFi HotStart Ready Mix, 12.5 ng of DNA obtained from the first-step PCR amplicon, and sterilised ultrapure water. The amplification programme included an initial denaturation step at 95 °C for 3 min, followed by 20 cycles of denaturation at 98 °C for 30 s, annealing at 55 °C for 30 s, and elongation at 72 °C for 30 s. The PCR products were purified using the FastGene^®^ Gel/PCR Extraction kit according to the manufacturer’s instructions.

For the third-step PCR, a primer set with flow cell adapter sequences, index sequences, and tailed sequences was used (forward primer, 5ʹ-AAT GAT ACG GCG ACC ACC GAG ATC TAC AC-Index sequence-TCG TCG GCA GCG TC-3ʹ, and reverse primer, 5ʹ-CAA GCA GAA GAC GGC ATA CGA GAT-Index sequence-GTC TCG TGG GCT CGG-3ʹ). The third-step PCR mixture (25 µl) comprised of 12.5 µl of Kapa HiFi HotStart Ready Mix, 0.5 µl of each primer (10 pM), and 11.5 µl of the second-step PCR amplicon. The amplification programme included an initial denaturation step at 95 °C for 3 min, followed by 8 cycles of denaturation at 98 °C for 30 s, annealing at 55 °C for 30 s, and elongation at 72 °C for 30 s. After electrophoresis in a 1.5% (w/v) agarose gel, the target bands were excised with sterilised cutters, and the DNA was extracted using the FastGene^®^ Gel/PCR Extraction kit, as described above. The DNA concentrations of the third-step PCR amplicons were quantified using a Qubit™ dsDNA HS Assay Kit (Thermo Fisher Scientific Inc.) according to the manufacturer’s instructions. The purified PCR products from each sample were mixed, denatured, and sequenced with a MiSeq System (Illumina) using the MiSeq Reagent Kit v3 (300 bp × 2 cycles with pair-end; Illumina), according to the manufacturer’s instructions. We obtained Good’s coverage values (> 95%) for all scalp hair shaft samples using the DNA extraction kit and PCR conditions described above. Good’s coverage values were estimated using QIIME™ 1.9.1 software^39^.

### Bioinformatics and statistical analysis

The index and universal sequences of each read were checked, and reads with complete index sequences were selected as valid sequences. USEARCH V8.1.1861^40^ software was used to merge paired-end reads and remove chimeric sequences. After the chimera check, the reads were grouped into operational taxonomic units (OTUs) with > 97% similarity. Alpha diversity (observed OTUs and Shannon index) was evaluated at a 1% OTU distance using the QIIME™ software package^39^. For taxonomy-based analysis, representative sequences of each OTU were analysed using the EzBioCloud platform^41^. PAM clustering was performed using the ‘pam’ function in the R library ‘cluster’, and the optimal number of clusters was chosen by maximising the Calinski–Harabasz index. The obtained cluster was validated using the silhouette index (http://cran.r-project.org/web/packages/cluster/index.html). The linear discriminant analysis effect size (LEfSe)^42^ method was used, via the Galaxy Browser, to detect significant differences in the relative abundance of the microbial taxa among clusters. SIMPER analysis was performed with the Bray Curtis dissimilarity metric using the R-package to examine which genera contributed to the differences among each extrinsic factor. Statistical analysis of bacterial cell number quantification was performed using ANOVA, and that of the bacterial community structure was performed using the Kruskal–Wallis test. Both analyses were performed using XLSTAT software ver. 2014 (http://www.xlstat.com/en/).

### Bioproject number

Illumina raw read sequences were deposited in the DDBJ/ENA/GenBank database under BioProject ID PRJDB11214.

## Supplementary Information

Supplementary Information.
