# Supplementary material for: Host factors that shape the bacterial community structure on scalp hair shaft
Source: Sci Rep. 2021 Sep 6;11:17711. doi: 10.1038/s41598-021-96767-w (PMC8421437; doi:10.1038/s41598-021-96767-w)
Supplement: Supplementary file 1 — Supplementary Information. [file 41598_2021_96767_MOESM1_ESM.pdf]

Title

Bacterial and host factors that shape the community structure on hair

Aurhors

Kota Watanabe[a], Azusa Yamada[a], Yuri Nishi[a], Yukihiro Tashiro[a,b*], Kenji Sakai[a,b*]

Author affiliation

[a]Laboratory of Soil and Environmental Microbiology, Division of Systems Bioengineering, Department of Bioscience and Biotechnology, Faculty of Agriculture, Graduate School, Kyushu University, Motooka 744, Nishi-ku, Fukuoka 819-0395, Japan

[b]Laboratory of Microbial Environmental Protection, Tropical Microbiology Unit, Center for International Education and Research of Agriculture, Faculty of Agriculture, Kyushu University, Motooka 744, Nishi-ku, Fukuoka 819-0395, Japan

Corresponding authors

Kenji Sakai

Laboratory of Soil and Environmental Microbiology, Division of Systems Bioengineering, Department of Bioscience and Biotechnology, Faculty of Agriculture, Graduate School, Kyushu University, Motooka 744, Nishi-ku, Fukuoka 819-0395, Japan.

TEL: +81-92-802-4738; E-mail: kensak@agr.kyushu-u.ac.jp

Yukihiro Tashiro

Laboratory of Soil and Environmental Microbiology, Division of Systems Bioengineering, Department of Bioscience and Biotechnology, Faculty of Agriculture, Graduate School, Kyushu University, Motooka 744, Nishi-ku, Fukuoka 819-0395, Japan.

TEL: +81-92-802-4739; E-mail: tashiro@agr.kyushu-u.ac.jp

Fig. S1 Correlation of bacterial cell number by qPCR and scalp hair characteristics.
(a) Correlation of bacterial cells / cm² and the hair length (cm). (b) Correlation of bacterial cells / cm² and the hair diameter.

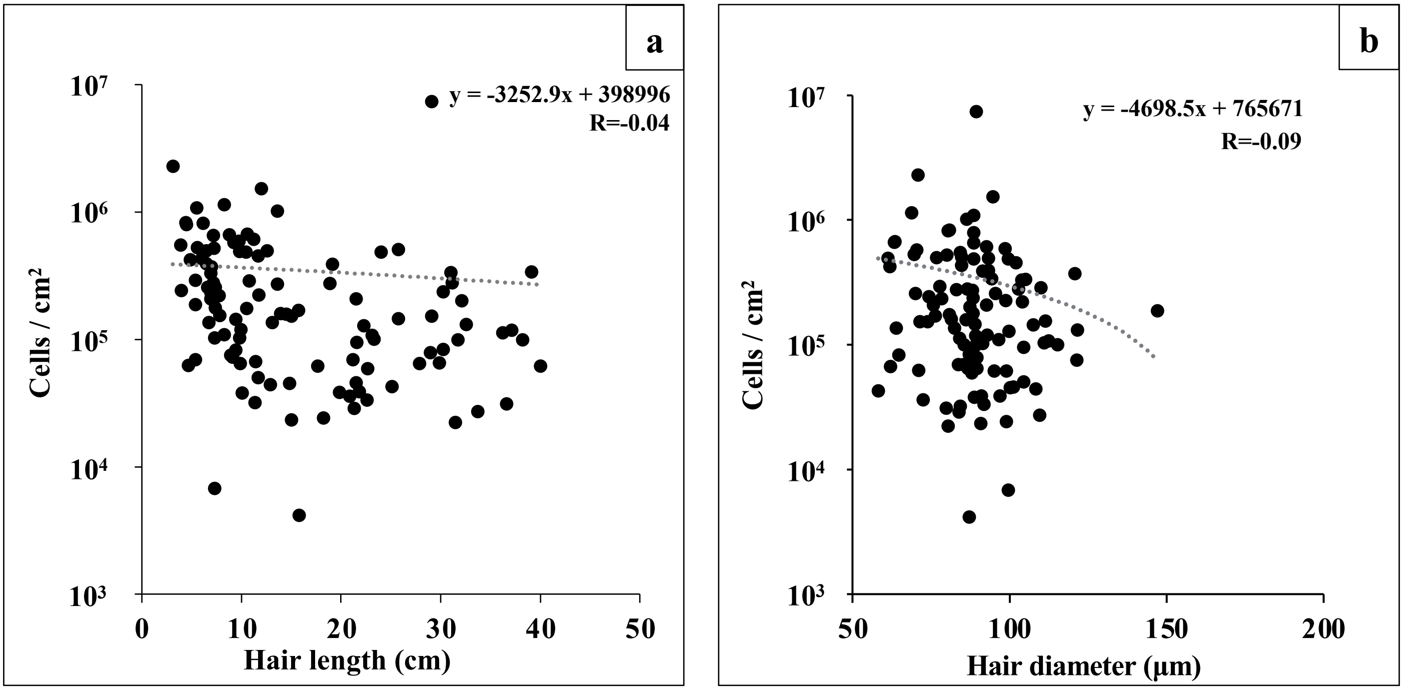

Table S1 Detailed information of 109 volunteers, quantification of bacterial copy number by qPCR of the bacterial 16S rRNA gene, and analysis of bacterial community structure by 16S amplicon sequencings.

No.	Gender	Age	Hair length (cm)	Hair diameter (μm)	Previous washed hair	Conditioner	Hair care product	Dryer	Wax	Hair color	Perm	Bleach
F001	Female	22	38.2	89.0	Lastnight	Use	Use	Use	Unused	Unused	Unused	Unused
F002	Female	22	10.6	63.3	Thismorning	Use	-	Unused	Unused	Use	Unused	Unused
F003	Female	21	25.8	88.9	Lastnight	Use	Use	Use	Unused	Use	Use	Unused
F004	Female	27	32.1	87.2	Lastnight	Unused	Unused	Use	Unused	Unused	Unused	Unused
F005	Female	23	21.5	75.6	Thismorning	Use	-	Use	Unused	Use	Use	Unused
F006	Female	22	29.1	73.9	Lastnight	Use	Use	Use	Unused	Use	Unused	Use
F007	Female	21	29	89.5	Lastnight	Unused	Use	Use	Unused	Use	Unused	Use
F008	Female	21	27.9	89.5	Thismorning	Use	Use	Use	Unused	Use	Unused	Unused
F009	Female	23	19.9	91.0	Lastnight	Use	Use	Use	Use	Use	Use	Unused
F010	Female	41	22.7	88.0	Lastnight	Use	Use	Unused	Use	Unused	Unused	Unused
F011	Female	27	36.7	79.7	Lastnight	Use	-	Use	Unused	Unused	Use	Unused
F012	Female	23	22.3	99.7	Lastnight	Use	Unused	Use	Unused	Use	Unused	Unused
F013	Female	36	30.3	87.1	Lastnight	Use	Use	Use	Unused	Use	Unused	Unused
F014	Female	43	32.6	121.5	Lastnight	Use	Use	Unused	Use	Use	Use	Unused
F015	Female	39	22.6	91.7	Lastnight	Use	Use	Use	Use	Use	Unused	Unused
F016	Female	39	19.2	91.3	Lastnight	Use	Use	Use	Unused	Unused	Use	Unused
F017	Female	24	13.1	63.8	Lastnight	Use	Unused	Use	Unused	Unused	Unused	Unused
F018	Female	23	13.9	81.3	Lastnight	Use	Unused	Use	Unused	Unused	Unused	Unused
F019	Female	23	40	95.0	Lastnight	Unused	Use	Use	Use	Use	Use	Unused
F020	Female	64	10.1	88.7	Lastnight	Use	Use	Use	Unused	Use	Unused	Unused
F021	Female	21	21.5	101.2	Lastnight	Use	Unused	Use	Use	Unused	Unused	Unused
F022	Female	22	17.7	98.9	Lastnight	Use	Use	Use	Unused	Use	Unused	Unused
F023	Female	37	14.5	86.0	Lastnight	Use	Unused	Use	Unused	Unused	Unused	Unused
F024	Female	21	21.6	104.4	Lastnight	Use	Use	Unused	Unused	-	-	Unused
F025	Female	26	15.1	90.8	Lastnight	Unused	Use	Use	Unused	Use	Unused	Unused
F026	Female	21	8.9	121.3	Lastnight	Use	Unused	Use	Unused	Unused	Use	Unused
F027	Female	20	14.9	100.0	Thismorning	Use	-	Use	Unused	Use	Unused	Use
F028	Female	20	11.7	104.4	Lastnight	Use	-	Use	Unused	Unused	Unused	Unused
F029	Female	19	9.8	93.3	Lastnight	Use	Use	Use	Use	Unused	Unused	Unused
F030	Female	22	7.35	88.3	Thismorning	Use	Unused	Use	Unused	Unused	Unused	Unused
F031	Female	19	23.1	112.2	Thismorning	Use	Unused	Use	Unused	Use	Unused	Unused
F032	Female	21	18.9	82.9	Lastnight	Use	Use	Use	Use	Use	Use	Unused
F033	Female	21	13.6	88.1	Lastnight	Use	Use	Use	Unused	Unused	Use	Unused
F034	Female	19	18.3	98.9	Lastnight	Use	Use	Use	Unused	Use	Unused	Unused
F035	Female	20	9.45	64.6	Lastnight	Unused	Use	Use	Use	Use	Unused	Unused
F036	Female	21	15	71.5	Lastnight	Use	Unused	Use	Use	Unused	Unused	Unused
F037	Female	21	11.4	84.1	Lastnight	Use	Unused	Use	Unused	Unused	Unused	Unused
F038	Female	19	21.9	96.8	Thismorning	Use	Unused	Use	Unused	Unused	Unused	Unused
F039	Female	22	21.4	83.9	-	Use	Use	Use	Use	Use	Unused	Use
F040	Female	22	25.8	84.6	Lastnight	Use	Use	Use	Unused	Use	Unused	Use
F041	Female	21	33.8	109.4	Thismorning	Use	Unused	Use	Use	Use	Unused	Unused
F042	Female	22	30.3	88.3	Lastnight	Use	Use	Use	Unused	Use	Unused	Use
F043	Female	21	20.9	72.3	Lastnight	Use	Use	Use	Use	Use	Unused	Use
F044	Female	24	39.1	94.2	Lastnight	Use	-	Unused	Unused	Unused	Unused	Unused
F045	Female	22	29.9	87.3	Lastnight	Unused	Unused	Use	Unused	Use	Unused	Use
F046	Female	20	11.4	62.0	Lastnight	Use	Use	Use	Use	Use	Unused	Unused
F047	Female	21	15.8	87.1	Lastnight	Use	Use	Use	Use	Use	Unused	Use
F048	Female	21	12	94.6	Lastnight	Use	-	Use	Unused	Unused	Unused	Unused
F049	Female	21	29.1	89.4	Lastnight	Use	Use	Use	Unused	Use	Unused	-
F050	Female	20	24	99.5	Lastnight	Unused	Use	Use	Unused	Use	Use	Unused
F051	Female	21	15.7	76.3	Lastnight	Use	-	Use	Use	Unused	Unused	Unused
F052	Female	20	31.8	85.5	Lastnight	Use	Use	Use	Use	Use	Unused	-
F053	Female	21	12.9	108.2	Lastnight	Use	Use	Use	Use	Use	Unused	Unused
F054	Female	21	25.1	58.2	Lastnight	Use	-	Use	Unused	Use	Use	Unused
F055	Female	20	9.85	86.7	Thismorning	Use	Unused	Use	Use	Use	Unused	Unused
F056	Female	21	31.2	102.7	Lastnight	Use	Unused	Use	Unused	Use	Unused	Unused
F057	Female	22	21.2	83.6	Lastnight	Use	Use	Use	Use	Use	Unused	Unused
F058	Female	24	31.5	80.3	Thismorning	Use	Unused	Use	Unused	Use	Unused	Unused
F059	Female	20	36.3	84.0	Lastnight	Use	Use	Use	Unused	Use	Unused	Unused
F060	Female	23	23.3	115.2	Lastnight	Use	Use	Use	Use	-	Unused	Unused
F061	Female	24	37.2	89.0	Lastnight	Use	Use	Use	Unused	Use	Unused	Unused
F062	Female	24	31	105.0	Lastnight	Use	Use	Use	Use	Use	Use	Unused
M001	Male	25	9.8	98.4	Thismorning	Unused	Unused	Use	Unused	Unused	Unused	Unused
M002	Male	26	3.1	70.8	Lastnight	Use	Unused	Use	Unused	Unused	Unused	Unused
M003	Male	22	8.3	68.7	Thismorning	Use	Unused	Use	Unused	Unused	Unused	Unused
M004	Male	22	6.5	76.6	Lastnight	Use	Unused	Use	Unused	Unused	Unused	Unused
M005	Male	22	11.7	102.0	Lastnight	Unused	Unused	Use	Unused	Unused	Use	Unused
M006	Male	29	10.8	109.8	Lastnight	Unused	Unused	Use	Unused	Unused	Unused	Unused
M007	Male	22	11.2	92.5	Lastnight	Unused	Unused	Use	Unused	Unused	Unused	Unused
M008	Male	23	10.5	88.4	Lastnight	Use	Unused	Use	Unused	Unused	Unused	Unused
M009	Male	25	5.5	88.4	Lastnight	Unused	Unused	Unused	Unused	Unused	Unused	Unused
M010	Male	22	10.5	80.8	Thismorning	Use	Unused	Use	Unused	Unused	Unused	Unused
M011	Male	23	10.0	92.8	Lastnight	Use	Unused	Unused	Unused	Unused	Unused	Unused
M012	Male	62	4.9	61.8	Lastnight	Unused	Unused	Unused	Unused	Unused	Unused	Unused
M013	Male	40	7.9	111.4	Lastnight	Unused	Unused	Use	Unused	Use	Unused	Unused
M014	Male	23	7.8	104.0	Thismorning	Unused	Unused	Use	Unused	Unused	Unused	Unused
M015	Male	31	7.3	99.4	Thismorning	Use	Unused	Use	Use	Unused	Unused	Unused
M016	Male	56	8.8	63.3	Thismorning	Use	Unused	Unused	Unused	Unused	Unused	Unused
M017	Male	37	6.2	80.4	Lastnight	Unused	Unused	Use	Unused	Unused	Unused	Unused
M018	Male	27	12.6	61.1	Thismorning	Unused	-	Use	Unused	Unused	Unused	Unused
M019	Male	27	6.6	95.3	Lastnight	Use	Unused	Unused	Unused	Unused	Unused	Unused
M020	Male	54	5.6	69.6	Lastnight	Unused	Unused	Unused	Use	Unused	Unused	Unused
M021	Male	45	6.9	78.3	Thismorning	Unused	Unused	Unused	Use	Unused	Unused	Unused
M022	Male	22	3.9	84.2	Lastnight	Unused	Unused	Use	Use	Unused	Unused	Unused
M023	Male	22	7.0	120.6	Lastnight	Use	Unused	Use	Unused	Unused	Unused	Unused
M024	Male	22	7.3	91.3	Thismorning	Unused	Unused	Use	Unused	Unused	Unused	Unused
M025	Male	33	11.8	98.8	Thismorning	Use	Unused	Unused	Unused	Use	Unused	Unused
M026	Male	21	4.7	71.0	Lastnight	Use	Unused	Use	Unused	Use	Unused	Unused
M027	Male	24	9.5	107.5	Thismorning	Unused	Unused	Use	Unused	Unused	Use	Unused
M028	Male	20	13.6	86.2	Thismorning	-	Unused	Use	Unused	Unused	Unused	Unused
M029	Male	21	5.4	77.6	Lastnight	Use	Unused	Use	Unused	Unused	Unused	Unused
M030	Male	20	6	84.7	Lastnight	Use	Unused	Use	Unused	Unused	Unused	Unused
M031	Male	22	9.8	110.8	Thismorning	Use	Use	Use	Unused	Unused	Unused	Unused
M032	Male	21	6.8	82.5	Thismorning	Unused	Unused	Use	Unused	Unused	Unused	Unused
M033	Male	20	7.3	79.9	Lastnight	Unused	Unused	Use	Unused	Unused	Unused	Unused
M034	Male	22	4.5	88.4	Lastnight	Unused	Unused	Use	Unused	Unused	Unused	Unused
M035	Male	20	4.4	80.8	Lastnight	Unused	Use	Use	Unused	Unused	Unused	Unused
M036	Male	38	7.2	86.5	Thismorning	Unused	Unused	Unused	Unused	Unused	Unused	Unused
M037	Male	25	6.5	93.2	Lastnight	Use	Unused	Use	Use	Unused	Unused	Unused
M038	Male	23	7.2	88.4	Lastnight	Use	Unused	Use	Use	Unused	Use	Unused
M039	Male	21	6.9	103.8	Lastnight	Use	Unused	Unused	Unused	Unused	Unused	Unused
M040	Male	20	4.0	74.3	Lastnight	Unused	Unused	Use	Use	Unused	Unused	Unused
M041	Male	21	8.3	96.4	Lastnight	Use	Unused	Use	Use	Use	Use	Unused
M042	Male	21	9.1	87.3	Lastnight	Unused	Use	Use	Use	Use	Unused	Unused
M043	Male	21	5.4	147.1	Lastnight	Unused	Unused	Use	Unused	Unused	Unused	Unused
M044	Male	20	7.4	69.9	Lastnight	Use	Unused	Use	Unused	Unused	Unused	Unused
M045	Male	20	5.4	85.2	Lastnight	Use	Unused	Use	Unused	Unused	Unused	Unused
M046	Male	22	6.9	92.7	Thismorning	Use	Unused	Use	Unused	Use	Use	Unused
M047	Male	24	9.3	70.3	Lastnight	Unused	Use	Use	Use	Unused	Unused	Unused

Table S2 Alpha diversity, total bacterial cells / cm², and absolute number of major bacterial genera at host intrinsic factors age and ethnicity.

Intrinsic factor	Factor type	Alpha-diversity		Total bacterial cells / cm^2 on scalp hair	bacterial cells / cm^2 on scalp hair				
		Observed OTUs	shannon		*Cutibacterium*	*Lawsonella*	*Pseudomonas*	*Moraxella*	*Staphylococcus*
Age	10-20s (n=93)	51.6(±17.3)	2.9(±0.7)	$3.6(\pm 8.1)\times 10^5$	$1.1(\pm 1.2)\times 10^5$	$3.8(\pm 7.5)\times 10^4$	$1.1(\pm 5.5)\times 10^5$	$2.2(\pm 6.0)\times 10^4$	$2.3(\pm 5.5)\times 10^4$
	30-40s (n=12)	46.7(±16.7)	2.9(±0.7)	$2.2(\pm 2.1)\times 10^5$	$8.8(\pm 7.3)\times 10^4$	$2.1(\pm 4.4)\times 10^4$	$7.1(\pm 12.5)\times 10^4$	$2.4(\pm 4.0)\times 10^3$	$9.9(\pm 9.2)\times 10^3$
	50-60s (n=4)	55.4(±4.3)	3.3(±0.2)	$4.1(\pm 2.3)\times 10^5$	$1.1(\pm 0.7)\times 10^5$	$5.8(\pm 5.0)\times 10^4$	$1.4(\pm 1.8)\times 10^5$	$1.5(\pm 2.1)\times 10^4$	$3.0(\pm 2.4)\times 10^4$
Etnicity	Japan (n=102)	52.0(±17.3)	2.9(±0.7)	$3.2(\pm 7.5)\times 10^5$	$1.0(\pm 1.1)\times 10^5$	$3.5(\pm 6.7)\times 10^4$	$9.1(\pm 51.6)\times 10^4$	$1.5(\pm 3.7)\times 10^4$	$2.3(\pm 5.3)\times 10^4$
	China (n=4)	36.4(±9.7)	2.7(±0.1)	$1.1(\pm 0.9)\times 10^6$	$2.7(\pm 2.6)\times 10^5$	$1.1(\pm 1.4)\times 10^5$	$5.1(\pm 4.9)\times 10^5$	$1.3(\pm 2.0)\times 10^5$	$6.0(\pm 2.4)\times 10^3$
	Thailand (n=2)	48.4(±17.2)	2.1(±0.7)	$1.9(\pm 0.3)\times 10^5$	$1.3(\pm 0.5)\times 10^5$	$4.0(\pm 2.5)\times 10^3$	$1.9(\pm 1.8)\times 10^4$	$1.1(\pm 0.2)\times 10^3$	$1.3(\pm 0.8)\times 10^4$
	America (n=1)	41	3.3	6.7×10^5	9.8×10^4	1.2×10^4	4.9×10^5	2.6×10^4	1.8×10^4

Table S3 Absolute number of major bacterial genera at each comparative category.

Extrinsic factor			Total bacterial cells/cm^2	bacterial cells / cm^2 on scalp hair				
				Cutibacterium	*Lawsonella*	*Pseudomonas*	*Moraxella*	*Staphylococcus*
Hair wash	Previous hair washed time	This morning (n=26)	$3.7(\pm 8.5)\times 10^5$	$8.1(\pm 6.7)\times 10^4$	$1.8(\pm 2.6)\times 10^4$	$9.8(\pm 18.5)\times 10^4$	$1.6(\pm 4.9)\times 10^4$	$1.1(\pm 1.1)\times 10^4$
		Last night (n=82)	$2.8(\pm 3.0)\times 10^5$	$1.1(\pm 1.3)\times 10^5$	$4.3(\pm 8.0)\times 10^4$	$1.1(\pm 5.8)\times 10^5$	$2.1(\pm 5.9)\times 10^4$	$2.6(\pm 5.8)\times 10^4$
	Conditioner	Use (n=77)	$3.3(\pm 8.8)\times 10^5$	$9.2(\pm 11.6)\times 10^4$ *	$2.5(\pm 5.0)\times 10^4$ *	$1.3(\pm 6.1)\times 10^5$	$1.7(\pm 5.7)\times 10^4$	$1.6(\pm 3.1)\times 10^4$ *
		Unused (n=31)	$3.7(\pm 2.7)\times 10^5$	$1.4(\pm 1.1)\times 10^5$	$6.8(\pm 10.3)\times 10^4$	$5.9(\pm 11.3)\times 10^4$	$1.7(\pm 3.6)\times 10^4$	$3.8(\pm 8.1)\times 10^4$
	Haircare	Use (n=40)	$3.5(\pm 10.1)\times 10^5$	$7.8(\pm 10.5)\times 10^4$ *	$2.5(\pm 6.7)\times 10^4$ *	$1.5(\pm 8.0)\times 10^5$	$1.2(\pm 2.6)\times 10^4$	$1.5(\pm 2.8)\times 10^4$ **
		Unused (n=59)	$3.4(\pm 3.7)\times 10^5$	$1.3(\pm 1.3)\times 10^5$	$4.9(\pm 7.8)\times 10^4$	$6.6(\pm 19.0)\times 10^4$	$1.9(\pm 4.3)\times 10^4$	$2.9(\pm 6.5)\times 10^4$
	Dryer	Use (n=94)	$3.4(\pm 8.1)\times 10^5$	$1.0(\pm 1.2)\times 10^5$	$3.4(\pm 6.4)\times 10^4$	$1.1(\pm 5.5)\times 10^5$	$2.1(\pm 6.0)\times 10^4$	$1.7(\pm 3.2)\times 10^4$
		Unused (n=15)	$3.6(\pm 2.7)\times 10^5$	$1.1(\pm 0.6)\times 10^5$	$5.6(\pm 10.4)\times 10^4$	$8.4(\pm 15.4)\times 10^4$	$9.2(\pm 13.3)\times 10^3$	$5.2(\pm 10.9)\times 10^4$
Hair styling	Wax	Use (n=32)	$1.8(\pm 1.9)\times 10^5$	$8.4(\pm 10.0)\times 10^4$	$1.9(\pm 3.2)\times 10^4$	$2.0(\pm 2.6)\times 10^4$	$1.3(\pm 3.0)\times 10^4$	$1.3(\pm 2.0)\times 10^4$
		Unused (n=77)	$4.2(\pm 8.8)\times 10^5$	$1.1(\pm 1.2)\times 10^5$	$4.5(\pm 8.2)\times 10^4$	$1.5(\pm 6.1)\times 10^5$	$2.2(\pm 6.4)\times 10^4$	$2.6(\pm 5.9)\times 10^4$
Chemical treatment	Color	Use (n=46)	$2.9(\pm 10.7)\times 10^5$	$6.4(\pm 9.0)\times 10^4$ **	$1.2(\pm 2.3)\times 10^4$	$1.4(\pm 7.5)\times 10^5$	$8.4(\pm 15.8)\times 10^3$	$1.1(\pm 2.3)\times 10^4$ **
		Unused (n=61)	$4.0(\pm 3.9)\times 10^5$	$1.4(\pm 1.3)\times 10^5$	$5.7(\pm 8.9)\times 10^4$	$8.6(\pm 21.1)\times 10^4$	$2.8(\pm 7.3)\times 10^4$	$3.1(\pm 6.4)\times 10^4$
	Perm	Use (n=18)	$2.3(\pm 1.7)\times 10^5$	$9.9(\pm 9.4)\times 10^4$	$3.1(\pm 4.6)\times 10^4$	$4.8(\pm 6.6)\times 10^4$	$1.1(\pm 1.6)\times 10^4$	$8.8(\pm 9.6)\times 10^3$
		Unused (n=90)	$3.7(\pm 8.3)\times 10^5$	$1.1(\pm 1.2)\times 10^5$	$3.8(\pm 7.6)\times 10^4$	$1.2(\pm 5.7)\times 10^5$	$2.1(\pm 6.1)\times 10^4$	$2.5(\pm 5.6)\times 10^4$
	Bleach	Use (n=9)	$1.3(\pm 1.5)\times 10^5$	$6.2(\pm 7.0)\times 10^4$	$1.4(\pm 2.0)\times 10^4$	$4.3(\pm 4.2)\times 10^3$	$1.5(\pm 2.5)\times 10^4$	$2.0(\pm 3.2)\times 10^4$
		Unused (n=98)	$3.7(\pm 8.3)\times 10^5$	$1.1(\pm 1.1)\times 10^5$	$3.8(\pm 7.4)\times 10^4$	$6.8(\pm 17.6)\times 10^4$	$2.0(\pm 5.9)\times 10^4$	$2.2(\pm 5.3)\times 10^4$

Kruslal-Wallis test, *p<0.05, **p<0.01

Table S4 Similarity percentage analysis of the genus differences with treatment. Contribution shows the dissimilarity explained by that genus.

	Extrinsic factor	Genus	Contribution	Mean abundance (This morning)	Mean abundance (Last night)	Change in abundance with treatment
Hair wash	Previous hair washed time	*Pseudomonas*	12.5%	23.0%	17.5%	5.5%
		Cutibacterium	12.2%	40.2%	42.3%	-2.2%
		Lawsonella	5.2%	9.4%	10.0%	-0.6%
		Staphylococcus	3.4%	5.3%	7.9%	-2.6%
		Moraxella	2.9%	4.2%	4.5%	-0.3%
Hair styling	Conditioner	*Cutibacterium*	11.2%	42.0%	41.3%	0.7%
		Pseudomonas	11.0%	20.5%	14.7%	5.9%
		Lawsonella	6.1%	8.1%	14.0%	-5.9%
		Staphylococcus	4.5%	6.2%	9.7%	-3.5%
		Moraxella	3.1%	4.4%	4.7%	-0.4%
	Haircare	*Pseudomonas*	11.6%	21.2%	17.4%	3.8%
		Cutibacterium	10.9%	43.8%	40.7%	3.1%
		Lawsonella	5.2%	7.7%	11.0%	-3.3%
		Staphylococcus	4.1%	6.6%	7.6%	-1.0%
		Moraxella	3.0%	4.1%	4.6%	-0.5%
	Dryer	*Pseudomonas*	11.8%	18.7%	19.4%	-0.7%
		Cutibacterium	11.7%	42.1%	39.7%	2.4%
		Lawsonella	5.5%	9.6%	11.2%	-1.6%
		Staphylococcus	4.3%	6.9%	9.5%	-2.6%
		Moraxella	2.6%	4.7%	2.9%	1.8%
	Wax	*Pseudomonas*	11.2%	19.6%	18.5%	1.1%
		Cutibacterium	11.0%	45.8%	40.2%	5.7%
		Lawsonella	4.9%	7.1%	11.0%	-3.9%
		Staphylococcus	4.1%	7.9%	7.0%	0.9%
		Moraxella	2.9%	3.9%	4.7%	-0.7%
Chemical treatment	Color	*Pseudomonas*	11.6%	22.3%	16.3%	6.0%
		Cutibacterium	10.9%	42.2%	41.5%	0.7%
		Lawsonella	5.3%	6.5%	12.2%	-5.7%
		Staphylococcus	4.0%	6.5%	7.8%	-1.3%
		Moraxella	2.9%	4.0%	4.8%	-0.9%
	Perm	*Pseudomonas*	11.6%	22.1%	18.2%	3.9%
		Cutibacterium	10.4%	42.9%	41.6%	1.3%
		Lawsonella	5.2%	10.0%	9.8%	0.2%
		Staphylococcus	3.3%	4.1%	7.9%	-3.7%
		Moraxella	2.7%	3.6%	4.6%	-1.0%
	Bleach	*Pseudomonas*	11.4%	15.8%	19.1%	-3.3%
		Cutibacterium	10.8%	45.4%	41.5%	3.9%
		Lawsonella	4.9%	8.7%	9.9%	-1.2%
		Staphylococcus	4.2%	8.2%	7.1%	1.1%
		Moraxella	3.4%	5.9%	4.3%	1.6%
